# Near-Patient Sampling to Assist Infection Control—A Case Report and Discussion

**DOI:** 10.3390/ijerph15020238

**Published:** 2018-01-31

**Authors:** Julian W. Tang, Elizabeth Hoyle, Sammy Moran, Manish Pareek

**Affiliations:** 1 Clinical Microbiology, University Hospitals of Leicester NHS Trust, Leicester LE1 5WW, UK; 2 Infection, Immunity and Inflammation, University of Leicester, Leicester LE1 7RH, UK; manish.pareek@leicester.ac.uk; 3 Infection Prevention and Control, University Hospitals of Leicester NHS Trust, Leicester LE1 5WW, UK; elizabeth.hoyle@uhl-tr.nhs.uk; 4 Leicester Children’s Hospital, University Hospitals of Leicester NHS Trust, Leicester LE1 5WW, UK; Sammy.Moran@uhl-tr.nhs.uk; 5 Infectious Diseases Unit, University Hospitals of Leicester NHS Trust, Leicester LE1 5WW, UK

**Keywords:** airborne, transmission, air sampling, respiratory, adenovirus, infection control, limit of detection, sensitivity, face masks, personal protective equipment

## Abstract

Air sampling as an aid to infection control is still in an experimental stage, as there is no consensus about which air samplers and pathogen detection methods should be used, and what thresholds of specific pathogens in specific exposed populations (staff, patients, or visitors) constitutes a true clinical risk. This case report used a button sampler, worn or held by staff or left free-standing in a fixed location, for environmental sampling around a child who was chronically infected by a respiratory adenovirus, to determine whether there was any risk of secondary adenovirus infection to the staff managing the patient. Despite multiple air samples taken on difference days, coinciding with high levels of adenovirus detectable in the child’s nasopharyngeal aspirates (NPAs), none of the air samples contained any detectable adenovirus DNA using a clinically validated diagnostic polymerase chain reaction (PCR) assay. Although highly sensitive, in-house PCR assays have been developed to detect airborne pathogen RNA/DNA, it is still unclear what level of specific pathogen RNA/DNA constitutes a true clinical risk. In this case, the absence of detectable airborne adenovirus DNA using a conventional diagnostic assay removed the requirement for staff to wear surgical masks and face visors when they entered the child’s room. No subsequent staff infections or outbreaks of adenovirus have so far been identified.

## 1. Introduction

Although air sampling has been extensively used in research studies [1,2,3,4,5] and in specific clinical situations (e.g., to assess the risk of aerosol-generating procedures—AGPs) [6,7], it is still not routinely used in hospital infection control situations, though this application is being explored currently by some teams [8,9].

There are several possible reasons for this: (i) despite there being a wide choice of air samplers either developed in-house or commercially available now [10,11,12,13,14], most infection control teams may not have access to one, and/or be familiar with its use outside of a specific research study context, and these air samplers are costly; (ii) the use of such air samplers requires a laboratory that can support its use, i.e., to be able to process such air samples in a routine manner. These may consist of air samples that have been collected into a fluid virus transport media (VTM) obtained from liquid impinger air samplers, or a gel disc (or other solid capture media) which can be dissolved in VTM. Testing would then usually be performed using molecular methods, mostly polymerase chain reaction (PCR) testing, with additional viral culture to confirm virus viability in the air sample.

However, most hospital diagnostic laboratories are automated and perform conventional, repetitive assays on human patient tissues or fluids. They do not have the training or manpower to run more complex research assays, especially those used to test air samples, which have to be more sensitive than conventional diagnostic assays; (iii) perhaps most importantly, the interpretation of these results is problematic, as there are no consensus guidelines as to what level of airborne pathogen constitutes a definitive infection control risk to patients, staff, or visitors. These thresholds are very likely to differ between the different patient populations for each pathogen (e.g., influenza on a bone marrow transplant versus respiratory syncytial virus on a general paediatric ward). Without this definitive, pathogen- and patient-specific risk threshold and guidance, it is very difficult for hospital infection control teams to apply the findings of and support such routine air sampling as part of their daily practice.

The other related, practical issue is then to define what the clinically relevant threshold should be for the limit of detection (LOD) of any molecular assay used to test such air samples. The following example illustrates some of these issues. Note that the figures discussed are for illustrative purposes only but the methods and equipment are based on some of the authors’ current experiments.

Typically, air samplers are switched on to sample at a specific airflow rate, e.g., at 12 L/min for the SKC biosampler (Cat No. 225 Series, SKC Ltd., Blandford Forum, UK), for a specific period of time (e.g., 1 h). Such time periods may be purely arbitrary and very often do not correlate with the actual durations of interaction between staff, patients, and visitors. Such air sampling will then draw a total air volume of: 12 L × 60 min = 720 L (or 720,000 cm^3^ = 0.72 m^3^) from the environment. For the SKC Biosampler, this amount of air will be sucked into a flask containing a swirling volume (20 mL) of VTM (e.g., Virocult, Medical Wire & Equipment, Corsham, UK) to capture any viruses present in the air. An aliquot of this 20 mL VTM can then be extracted and tested using PCR to detect any virus present.

If our pathogen of interest is respiratory syncytial virus (RSV), for example, and our assay can detect down to a minimum of ~1000 DNA copies/mL VTM (as the reported LOD for the AusDiagnostics respiratory virus PCR assay below), how can we interpret and risk-assess any result from this air sample? When air sampling in a general practitioner (family doctor) clinic or a hospital paediatric ward, during the autumn or winter, it may not necessarily be unusual to detect some respiratory viruses, such as RSV [5].

To continue our illustrative example, after back-calculating the airborne viral load from the specific PCR reaction volumes, let us assume that 2000 RSV RNA copies was detected from the air sample collected by the SKC biosampler, i.e., representing 2000 copies of RSV RNA from the 20 mL VTM total volume in the SKC biosampler. As this has resulted from the 0.72 m^3^ of sampled air, this equates to 2778 copies/m^3^ air of RSV RNA.

Does this level of RSV RNA in the air pose a risk to patients, staff or visitors in the vicinity? More generally speaking, at what level of any airborne virus detection, should we become concerned and intervene? This answer is clearly very context-dependent, and depends on the immune status of those exposed, which in turn will partially determine the pathogen’s specific infectious dose (i.e., the amount of the organism required to cause infection, and potentially, disease). This will also vary between individuals depending on their past exposure and current level of specific immunity to that specific pathogen, including any history of vaccination, where available. Another layer of complexity is posed by the use of PCR testing of the air sample, as PCR cannot distinguish between live and inactivated virus. Any individual infectious dose estimate for a specific pathogen for a specific exposed population will need to take this into account.

Finally, even if we knew all of the above, we do not actually know exactly how much air volume needs to be inhaled by a specific individual to achieve that infectious dose. To make calculations simple, we would have to assume a uniform distribution of the airborne pathogen within that air volume and then calculate the volume of air required to be inhaled to achieve that infectious dose. Furthermore, it is unclear how much of that inhaled air is either re-exhaled [15], or how much contact the airborne pathogen will have with its corresponding host cell receptors (i.e., for viruses) which may be distributed differently within the respiratory tract in different individuals [16,17].

Working this into our example above, if the infectious dose for RSV is about 2000 RSV RNA copies/m^3^ air for a specific exposed population, but we know from previous experiments that of our detected 2778 copies/m^3^ RSV RNA, only 30% (i.e., 833 RSV RNA copies/m^3^) of this detected RSV is viable then it is unlikely that any secondary cases of RSV will occur, as the airborne infectious dose of RSV has not been reached.

The following case report, describing a child with a chronic respiratory adenovirus (AdV) infection, highlights several of the points raised above. Ultimately, in this case, these results, together with an understanding of the closed nature of the tracheostomy system, did help to give confidence to the staff managing this patient that they were not being exposed to clinically significant levels of adenovirus in the air. Taken together, these findings allowed the staff to remove their face masks, whilst maintaining routine contact transmission precautions, to enhance the social interaction and development of this young patient.

## 2. Case Report

A tetraplegic, bed-bound, young child with an underlying congenital immunodeficiency, on long-term ventilator support via a tracheostomy, became chronically infected with a seasonal respiratory adenovirus (AdV, serotype C1). Regular nasopharyngeal aspirates (NPAs) showed AdV loads varying from 1000 copies/mL to 163 million copies/mL using a quantitative AdV DNA PCR assay over a four-month period.

Due to some of this high level of respiratory AdV being potentially shed into the environment (i.e., into the air and onto surfaces) from patient’s respiratory tract, the hospital Infection Prevention and Control (IPC) team required all healthcare staff to wear personal protective equipment (PPE) when managing this patient. This consisted of donning surgical masks, face visors, gloves, and gowns, each time they entered the room, whether it was for physiotherapy, educational and play periods, as well as the daily maintenance of her tracheostomy.

The paediatric team were therefore concerned whether this would affect the child’s psycho-social development as only the staffs’ eyes but no other facial expressions were visible to the child, each time they visited.

Near-patient air sampling was initiated to assess the actual exposure to staff of clinically significant, airborne levels of AdV potentially produced by this patient (e.g., via air leakage around the tracheostomy cuff), to determine whether such a high level of PPE was necessary.

## 3. Materials and Methods

An SKC button sampler with gel filters, powered by an AirChek XR5000 pump and calibrated by a rotameter (all supplied by SKC Ltd., Blandford Forum, UK) was used for all the air sampling.

**Figure 1:**
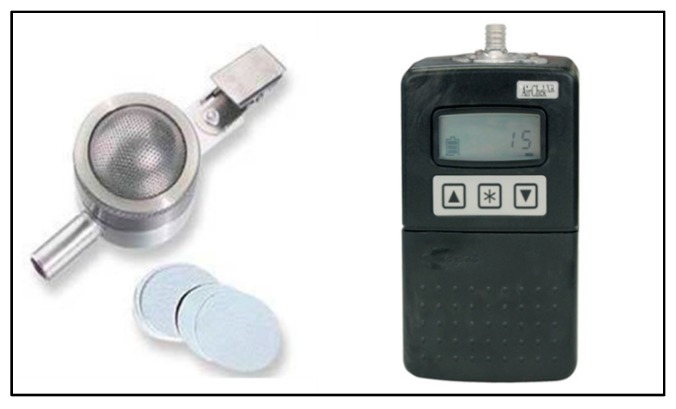
Portable (wearable) SKC Button Sampler (left) connected to the AirChek XR5000 pump (right) as used for near-patient air sampling in this study. The manufacturer’s sampling rate was fixed at 4 L/min. (Available from http://www.skcltd.com/index.php/uncategorised-articles/212-bio-aerosol-sampling-products).

This sampler was used in one of three ways: it was worn by ward-based staff (nurses, physiotherapists, or education and play assistants), held approximately 0.2–0.3 m away from the patient’s nose and mouth, or carefully mounted and left standing overnight in a fixed location near the patient, to sample ambient air in the vicinity. The air was drawn through the dissolvable gel filter at an airflow rate of 4 L/min. The pump was calibrated to 4 L/min with the rotameter prior to each sampling session (Figure 1). Local room temperature and relative humidity was also measured at the start of each air sampling session (ThermoPro TP50, My iTronics, Shenzhen, Guangdong, China).

At the end of the sampling period, the gel filter was carefully extracted from the filter, dissolved in 2 mL of virus transport medium (VTM), extracted (QIAsymphony DSP Virus/Pathogen Kit, Qiagen, Manchester, UK) and run on the AusDiagnostics (Respiratory Viruses 16-WELL, cat. 20602, AusDiagnostics UK Ltd., Chesham, UK) assay. This commercial assay has a claimed lower limit of detection of 1075 AdV DNA copies/mL. This same assay was also used for the weekly testing of NPAs and endotracheal tube aspirates (ETAs) from this same patient to monitor the progress of the chronic AdV infection. All positive AdV NPA samples were sent to a commercial laboratory for AdV typing and quantification (Micropathology Ltd., Coventry, UK).

**Figure 2:**
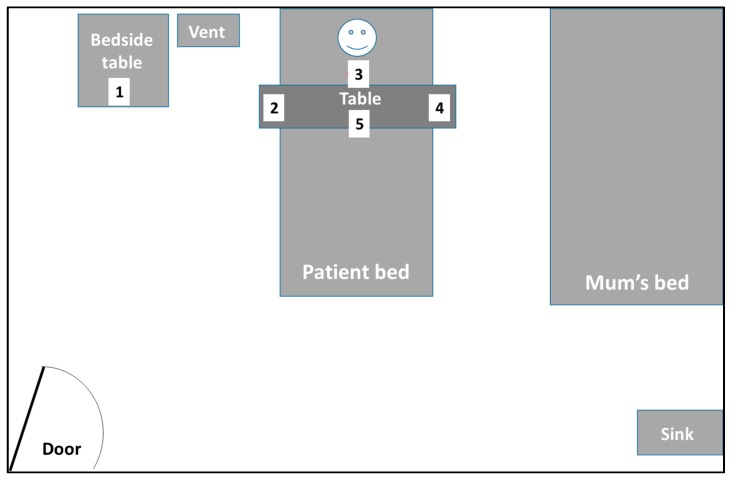
Patient room layout and air sampling sites and durations during various activities for the patient. Position 1—environmental air sampling; 2—during daily suctioning of the tracheostomy (worn by physiotherapist); 3—hand-held over tracheostomy site; 4—during an educational session (worn by educational facilitator); 5—overnight environmental air sampling. See main text (Section 4) for more details.

Various positions (most commonly frequented by staff during routine care duties) around the patient were sampled for varying durations, multiple times, over 3–4 weeks, to check for the presence of airborne AdV (Figure 2). The air sampling sessions were coordinated to coincide with the days on which routine NPAs were taken from the patient for monitoring the AdV levels. In addition to the NPAs, ETAs were also routinely taken for respiratory virus PCR testing to check for the presence of any AdV in the lower respiratory tract (LRT).

Between each air sampling session, the button sampler and accompanying tubing were thoroughly washed with warm soapy water, rinsed, then air-dried to physically remove any respiratory virus nucleic acid that might be present on the inside surfaces. ‘Dry’ runs using just the gel filter dissolved in VTM failed to detect any AdV DNA. This VTM was also used on the wards for daily routine clinical sampling, and as a negative extraction and PCR control during normal diagnostic testing, so any potential for viral contamination was monitored on a daily basis.

After discussion with our local institutional review board (IRB) it was agreed that this air sampling constituted environmental sampling and therefore did not require a formal ethics approval.

## 4. Results

**Figure 3:**
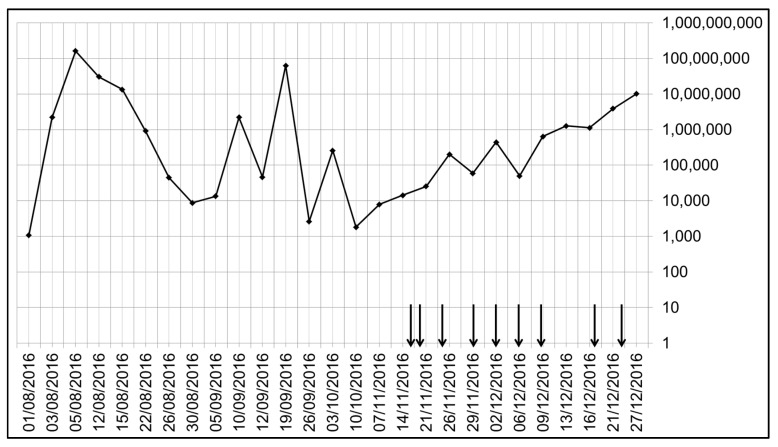
Variable AdV DNA levels in the routine patients nasopharyngeal aspirates (NPAs). Note that the date increases from left to right along the *x*-axis, and the *y*-axis (placed on the right-hand side to allow easier comparison with dates of sampling) shows the AdV DNA in copies/mL VTM. Air sampling was performed on the dates indicated by the vertical black arrows. Despite high NPA AdV DNA levels at times, no AdV DNA was detected in any of the air samples with the same routine commercial diagnostic respiratory PCR assay (spiked samples with positive AdV DNA extract showed no inhibition from the gel filter).

In total nine air samples were taken over a period of one month. The results have been summarised in Table 1. From Figure 3, it can be seen that even in the three months prior to the air sampling study period, the levels of AdV DNA in the routine NPA samples varied dramatically over a range of 1000 to 100 million (i.e., over 5 log_10_) AdV DNA copies/mL. During the air sampling period, this variation was more limited (from 10,000 to 10 million AdV DNA copies/mL). This large variation and lack of viral clearance was likely due to the establishment of some degree of AdV latency in the adenoids with ongoing reactivation in a child with an underlying primary (congenital) immunodeficiency.

Despite the varying and occasionally very high levels of AdV DNA levels in the corresponding NPA samples, no AdV DNA was detected in any of the air samples. In addition, none of the ETA samples were positive for AdV DNA.

As well as the negative controls described in the Methods section, further control experiments involving spiking the gel filters prior to extraction and PCR with the patient’s positive AdV NPA samples showed no inhibition. There was minimal (1 Ct or less) impact of the dilution factor due to the gel filter being dissolved in 2 mL VTM compared to the neat NPA PCR signal.

This indicates that during this study period, despite these high levels of AdV present in the patient’s nasal and oropharyngeal cavities, very little if any virus was being released into the air around the patient’s breathing zone whilst the tracheostomy was in situ.

## 5. Discussion

This study has several limitations but also raises interesting questions. The SKC button sampler is a personal, portable, low volume sampler. The duration of sampling when worn by the ward staff was typically relatively short, usually around 30–40 min to match the actual periods of interaction with the patient. During this time, the sampler would have sampled (at 4 L/min) 120–160 L of air. This is slightly less but comparable to the amount of air that might be inhaled by one of the ward nurses working in the same vicinity of the patient. This estimate is based on a tidal volume of 0.5–0.7 L and a respiratory rate of 12–16 breaths per minute (bpm), which gives a total inhaled volume of approximately 180–336 L over 30 min, depending on their activity level.

It is known that air samples are very low in pathogen genome concentration, which is a general problem with analysing air samples in metagenomic studies [18]. Thus, the use of a routine clinical diagnostic assay for testing air samples may be deemed insufficiently sensitive for this purpose, as such assays are indeed optimised for the testing of clinical samples, such as nasal/throat swabs, NPAs, ETAs or broncho-alveolar lavages (BALs), where the pathogen concentrations are generally higher. One future option to increase the sensitivity of such assays for air samples would be to increase volume of VTM sample (containing the dissolved gel filter) input into the extraction step. However, for this study, we deliberately intended to test the sensitivity of the standard diagnostic respiratory PCR assay protocol to detect airborne virus.

This then poses the question what level of detection constitutes a clinical risk? That is, what detectable level of airborne pathogen is sufficient to cause infections in nearby susceptible hosts? Would more sensitive assays help if they detect airborne levels of pathogen below such a threshold? If these lower levels of airborne pathogen do not pose a clinical risk, then is it useful to use an assay that detects such low levels of airborne pathogen, when no intervention will result from this? The use of a desensitised assay in other areas of diagnostic virology is well accepted, e.g., for CMV viral load testing in pre-emptive therapy, post-bone marrow transplant, when overly sensitive CMV PCR assays would lead to unnecessary treatment (which itself can have severe adverse effects). Furthermore, what more sensitive assays can a routine diagnostic laboratory be expected and be capable of performing for such air samples?

Although several studies have performed near-patient air sampling [19,20,21], this question has generally not been well-investigated nor answered for common everyday pathogens in a routine clinical environment to date. One estimate for the aerosol/airborne infectious dose of influenza is about 1950–3000 copies of viable virus [22]. Older estimates have been reported for some bioterrorism agents [23]. So although more sensitive assays may detect fewer copies of pathogen nucleic acid in air samples, the actual clinical risk such lower detectable levels of virus pose are still unclear.

In this patient, the fact that all the ETAs (i.e., the deeper lung samples) showed undetectable levels of AdV DNA suggests that the AdV was only present in the upper respiratory tract (URT), where it had probably become latent in her adenoids. In theory, any AdV in this region was unlikely to be expelled because any airflow from deeper in her lungs would be channelled into her tracheostomy. The tracheostomy cuff around the tube, holding it in place in her trachea, should also prevent any escape of air into her URT. So even if AdV is being reactivated and shed from her adenoids in the URT, there would be very little air entering the URT from her lungs to aerosolise and exhale it into the surrounding air. Thus, in theory, any AdV would be purely confined to the patient’s mouth and saliva and not become airborne. The air sampling was thus performed to confirm this theory, i.e., that no clinically significant amount of AdV was exhaled into the surrounding air, *in the presence of the tracheostomy*.

However, it is important to note (and so that this is not misunderstood by infection control teams) that given the above discussion over the lack of sensitivity of conventional diagnostic respiratory virus PCR assays for the detection of viral nucleic acid in air samples, these air sampling results *alone* may not have been sufficient to allow the staff to remove their respiratory protection.

Hence, the negative air sampling results *in conjunction with the closed tracheostomy system that was in place*, provided *additional* confidence to the clinical care teams that no clinically significant levels of AdV were being aerosolised into the environment, which might potentially lead to nosocomial staff infections through direct inhalation, or via self-inoculation from aerosol-contaminated fomites [24]. This allowed the staff to enter the room without the need to wear surgical masks and face visors. So far, there have been no resulting adenovirus infections or outbreaks reported amongst the staff on this ward.

## 6. Conclusions

Although infection prevention and control guidelines are necessarily precautionary and generally universal to avoid confusion, supplemental near-patient air sampling can sometimes be used to refine or reassure the need for PPE requirements in specific patient cases, where the need arises.

## Figures and Tables

**Table 1:** Summary of air sampling results. Relative humidity range: 32% to 46%. Temperature range: 23.4 °C to 24.8 °C.

Sampling Position (with Reference to Figure 1)	Number of Samples Taken	Distance from Patient’s Head (m) ^1^	Sampling Duration (min)	Volume of Air Sampled (L)	Corresponding NPA AdV Level (DNA Copies/mL) ^2^	Amount of AdV DNA Detected in Air Sample
1 (bedside table, to the right of the patient)	2	1.0–1.5	338	1352	14,300–25,200	None
			248	992	14,300–25,200	
2 (on right side of patient during suctioning of tracheostomy—sampler worn by physiotherapist on collar)	2	0.5–1.0	26	104	943,000	None
			45	180	58,800	
3 (held by lead author, JWT, over tracheostomy site, during suctioning by physiotherapist)	3	0.2–0.3	18	72	440,000	None
			29	116	49,900	
			28	112	1.13 × 10^6^	
4 (on the left side of the patient during an educational session—sampler worn by educational therapist on collar)	1	0.3–0.5	38	152	25,200–201,000	None
5 (overnight sampling whilst patient was sleeping—positioned on table over patient bed)	1	0.3–0.5	879	3516	3.88 × 10^6^–10.1 × 10^6^	None

^1^ range given as some staff movement is normal during these activities; ^2^ range given where the NPA sampling dates fall on either side of the air sampling dates; NPA—nasopharyngeal aspirate; AdV—adenovirus.

## References

[1] Booth T.F., Kournikakis B., Bastien N., Ho J., Kobasa D., Stadnyk L., Li Y., Spence M., Paton S., Henry B. (2005). Detection of airborne severe acute respiratory syndrome (SARS) coronavirus and environmental contamination in SARS outbreak units. J. Infect. Dis..

[2] Matuka O., Singh T.S., Bryce E., Yassi A., Kgasha O., Zungu M., Kyaw K., Malotle M., Renton K., O’Hara L. (2015). Pilot study to detect airborne Mycobacterium tuberculosis exposure in a South African public healthcare facility outpatient clinic. J. Hosp. Infect..

[3] Lindsley W.G., Blachere F.M., Davis K.A., Pearce T.A., Fisher M.A., Khakoo R., Davis S.M., Rogers M.E., Thewlis R.E., Posada J.A. (2010). Distribution of airborne influenza virus and respiratory syncytial virus in an urgent care medical clinic. Clin. Infect. Dis..

[4] Milton D.K., Fabian M.P., Cowling B.J., Grantham M.L., McDevitt J.J. (2013). Influenza virus aerosols in human exhaled breath: Particle size, culturability, and effect of surgical masks. PLoS Pathog..

[5] Kulkarni H., Smith C.M., Lee D.D.H., Hirst R.A., Easton A.J., O’Callaghan C. (2016). Evidence of Respiratory Syncytial Virus Spread by Aerosol. Time to Revisit Infection Control Strategies?. Am. J. Respir. Crit. Care Med..

[6] O’Neil C.A., Li J., Leavey A., Wang Y., Hink M., Wallace M., Biswas P., Burnham C.D., Babcock H.M. (2017). Centers for Disease Control and Prevention Epicenters Program: Characterization of Aerosols Generated During Patient Care Activities. Clin. Infect. Dis..

[7] Thompson K.A., Pappachan J.V., Bennett A.M., Mittal H., Macken S., Dove B.K., Nguyen-Van-Tam J.S., Copley V.R., O’Brien S., Hoffman P. (2013). EASE Study Consortium: Influenza aerosols in UK hospitals during the H1N1 (2009) pandemic—The risk of aerosol generation during medical procedures. PLoS ONE.

[8] Nguyen T.T., Poh M.K., Low J., Kalimuddin S., Thoon K.C., Ng W.C., Anderson B.D., Gray G.C. (2016). Bioaerosol Sampling in Clinical Settings: A Promising, Noninvasive Approach for Detecting Respiratory Viruses. Open Forum Infectious Diseases.

[9] Ladhani L., Pardon G., Meeuws H., van Wesenbeeck L., Schmidt K., Stuyver L., van der Wijngaart W. (2017). Sampling and detection of airborne influenza virus towards point-of-care applications. PLoS ONE.

[10] Fabian P., McDevitt J.J., Houseman E.A., Milton D.K. (2009). Airborne influenza virus detection with four aerosol samplers using molecular and infectivity assays: Considerations for a new infectious virus aerosol sampler. Indoor Air..

[11] Blachere F.M., Cao G., Lindsley W.G., Noti J.D., Beezhold D.H. (2011). Enhanced detection of infectious airborne influenza virus. J. Virol. Methods.

[12] Wang C.H., Chen B.T., Han B.C., Liu A.C., Hung P.C., Chen C.Y., Chao H.J. (2015). Field evaluation of personal sampling methods for multiple bioaerosols. PLoS ONE.

[13] L’Orange C., Anderson K., Sleeth D., Anthony T.R., Volckens J. (2016). A Simple and Disposable Sampler for Inhalable Aerosol. Ann. Occup. Hyg..

[14] Uhrbrand K., Koponen I.K., Schultz A.C., Madsen A.M. (2017). Evaluation of air samplers and filter materials for collection and recovery of airborne norovirus. J. Appl. Microbiol..

[15] Wei J.J., Tang J.W., Borojeni A.A.T., Yin S., Martin M., Finlay W.H., Li Y. (2016). Low re-inhalation of the exhaled flow during normal nasal breathing in a pediatric airway replica. Build. Environ..

[16] Nicholls J.M., Chan R.W., Russell R.J., Air G.M., Peiris J.S. (2008). Evolving complexities of influenza virus and its receptors. Trends Microbiol..

[17] Barlan A., Zhao J., Sarkar M.K., Li K., McCray P.B., Perlman S., Gallagher T. (2014). Receptor variation and susceptibility to Middle East respiratory syndrome coronavirus infection. J. Virol..

[18] Behzad H., Gojobori T., Mineta K. (2015). Challenges and opportunities of airborne metagenomics. Genome Biol. Evol..

[19] Bischoff W.E., Swett K., Leng I., Peters T.R. (2013). Exposure to influenza virus aerosols during routine patient care. J. Infect. Dis..

[20] Cummings K.J., Martin S.B., Lindsley W.G., Othumpangat S., Blachere F.M., Noti J.D., Beezhold D.H., Roidad N., Parker J.E., Weissman D.N. (2014). Exposure to influenza virus aerosols in the hospital setting: Is routine patient care an aerosol generating procedure?. J. Infect. Dis..

[21] Hatagishi E., Okamoto M., Ohmiya S., Yano H., Hori T., Saito W., Miki H., Suzuki Y., Saito R., Yamamoto T. (2014). Establishment and clinical applications of a portable system for capturing influenza viruses released through coughing. PLoS ONE.

[22] Nikitin N., Petrova E., Trifonova E., Karpova O. (2014). Influenza virus aerosols in the air and their infectiousness. Adv. Virol..

[23] Franz D.R., Jahrling P.B., Friedlander A.M., McClain D.J., Hoover D.L., Bryne W.R., Pavlin J.A., Christopher G.W., Eitzen E.M. (1997). Clinical recognition and management of patients exposed to biological warfare agents. JAMA.

[24] Hoyle E., Erez J.C., Kirk-Granger H.R., Collins E., Tang J.W. (2016). An adenovirus 4 outbreak amongst staff in a pediatric ward manifesting as keratoconjunctivitis—A possible failure of contact and aerosol infection control. Am. J. Infect. Control.

